# Factors Associated with Perceived Racial Discrimination While Receiving Medical Care in the United States

**DOI:** 10.3390/healthcare13151906

**Published:** 2025-08-05

**Authors:** Elizabeth Ayangunna, Kingsley Kalu, Bushra Shah, Indira Karibayeva, Gulzar Shah

**Affiliations:** 1 Independent Researchers, Statesboro, GA 30460, USA; ea06435@georgiasouthern.edu (E.A.); kk13870@georgiasouthern.edu (K.K.); 2 Department of Health Policy and Community Health, Jiann-Ping Hsu College of Public Health, Georgia Southern University, Statesboro, GA 30460, USA; bs06779@georgiasouthern.edu (B.S.); ik01379@georgiasouthern.edu (I.K.)

**Keywords:** perceived racial discrimination, racial disparities, healthcare access, health equity, structural racism, social determinants of health, cross-sectional study, United States, HINTS survey, logistic regression

## Abstract

**Background:** Health equity can only be achieved when every individual has access to quality healthcare without fear of being discriminated against. This study analyzed the sociodemographic characteristics associated with self-reported racial discrimination when receiving medical care in the United States. **Methods:** This quantitative cross-sectional study utilized the 2022 National Trends Survey 6. We performed a logistic regression analysis using 6102 survey responses from study participants who answered the question about perceived discrimination. **Results:** Older adults aged 75 years and above had significantly lower odds of reporting perceived discrimination when receiving medical care compared to those aged 18–34 years (AOR = 0.24; 95% CI: 0.10–0.58). The odds of reporting perceived discrimination were significantly higher among non-Hispanic Blacks (AOR = 7.30; 95% CI: 4.48–11.88), Hispanics (AOR = 3.56; 95% CI: 2.45–5.17), non-Hispanic Asians (AOR = 5.95; 95% CI: 2.25–15.73), and individuals identifying as non-Hispanic Other (AOR = 10.91; 95% CI: 5.42–21.98), compared to non-Hispanic Whites. Compared to individuals from households earning less than USD 20,000, the odds of reporting perceived discrimination when receiving medical care were significantly lower among individuals from households earning between USD 50,000 and <USD 75,000 (AOR = 0.42; 95% CI: 0.23–0.78) and those earning USD 75,000 or more (AOR = 0.43; 95% CI: 0.22–0.83). **Conclusions:** Despite having a multicultural and ethnically diverse population, racial discrimination persists in the United States and has become a barrier to achieving health equity. Health organizations should implement policies that ensure health workers attend mandatory anti-racism training.

## 1. Introduction

Discrimination is the unjust or prejudicial treatment of an individual or group of people based on their race, age, gender, or sexual orientation [1]. Although discrimination is sometimes used interchangeably with racism, racism is systemic oppression that unfairly treats individuals or groups of people due to their race or ethnicity [2,3,4]. Racism is bolstered by discrimination and can be structural, interpersonal, and institutional. In a seminal review, Gee and Ford (2011) highlight various forms of structural racism, such as residential segregation, immigration policies, and intergenerational inequities, and their detrimental effects on the health of marginalized populations [5]. Racism relies on a power dynamic in which the dominant group has access to more privileges and resources. Racism in the United States is a very complex issue with multiple contributing factors, including passivism, media, power reinforced by legislation, and segregation [2,3,4].

Discrimination affects health by adversely impacting individuals’ living conditions, housing, employment, education, and differences in healthcare quality and access [6]. Overall, discrimination as a Social Determinant of Health (SDOH) encompasses different levels of discrimination, such as individual, racial, and structural discrimination, which are linked to health inequities [7,8]. Individual discrimination is described as the unfair interaction between personal characteristics, such as sex and race, and institutional roles, including those of a patient and healthcare provider, or as a private or public individual [2,4,6,7,8]. Structural discrimination refers to social forces, macro-level systems, ideologies, institutions, and interconnected processes that reinforce and generate inequities [7].

Racial discrimination is the most common form, followed by discrimination based on gender, age, income, and education [9]. Racial discrimination can be defined as the “unfair treatment that members of marginalized racial and ethnic groups experience because of their phenotypical or linguistic characteristics and cultural practices” [10]. Racial discrimination has been linked to adverse health outcomes, including poor physical health—such as hypertension, increased risk of cardiovascular diseases, obesity, breast cancer, and elevated all-cause mortality—as well as poor mental health, including anxiety, depression, substance abuse, and psychological distress, ultimately contributing to a significant reduction in life expectancy [11,12,13,14,15,16]. Racial discrimination within the healthcare system is a public health issue. In the healthcare system, racial discrimination creates a lack of consideration or prejudicial treatment due to a preconceived notion about the identity of an individual or group of people, posing a severe barrier to equitable healthcare, poor quality of care, and adverse health outcomes [17,18]. A recent study revealed that approximately 20% of adults in the United States have experienced some form of discrimination while receiving healthcare—most of which was racially related—highlighting the persistence of discrimination despite the country’s status as one of the world’s most multicultural and ethnically diverse nations, with a population of about 335 million as of 2024 [19,20,21,22]. Past studies show that a large percentage of United States healthcare workers have witnessed discrimination against patients and confirm that patients may receive discrepant care based on their ethnicity, race, or language [17,23].

Racial discrimination within the healthcare system is not an isolated phenomenon but a manifestation of more profound structural inequities. These individual experiences of discrimination are embedded within and perpetuated by broader systemic policies and practices that systematically disadvantage racial and ethnic minorities, thereby reinforcing structural racism. For instance, discriminatory practices in healthcare settings, such as implicit bias among providers or unequal access to quality care, are often rooted in historical and institutionalized policies that marginalize certain groups, leading to persistent health disparities [24,25]. One of the core drivers of health inequalities at the individual level and in certain groups and communities stems from structural racism, and when reinforced by racial discrimination while receiving medical care, it can lead to inequitable access to public health and healthcare services [26].

Numerous studies have shown that the experiences of discrimination encountered within the healthcare system can adversely impact patients’ trust and health-seeking behaviors regarding healthcare utilization [27,28,29].

Despite government policies and public health interventions in the healthcare system, racial discrimination when receiving medical care is still a public health problem [30,31,32]. Several studies have focused on racial discrimination, including examining how perceived discrimination was associated with mortality among a bi-racial sample population, the experience and perspective on discrimination among African American adults, and the unfair judgment in healthcare among these sub-groups [33,34,35]. Nong et al. (2020) examined different types of discrimination reported by patients within the United States’ healthcare system, concluding that discrimination in healthcare was more frequent than previously documented in studies [9]. Another study shed light on the concept of discrimination, diversity, and their impact on healthcare, arguing that racial discrimination disproportionately impacts people of color and harms patients due to fear of negative stereotypes [18].

Although studies have shown that people of color are disproportionately affected by racial discrimination when receiving medical care, this study provides updated information on sociodemographic characteristics, including race, associated with this public health problem, using data collected in the post-pandemic period. This study aimed to identify sociodemographic factors associated with perceived racial discrimination when receiving medical care in the United States. For this study, the authors defined perceived racial discrimination as self-reported discrimination or unfair treatment received during medical care because of race or ethnicity. Understanding these characteristics using a nationally representative sample is crucial for developing effective policies and interventions.

## 2. Materials and Methods

### 2.1. Data Source

This quantitative cross-sectional study utilized secondary data from the 2022 Health Information National Trends Survey (HINTS) iteration. Specifically, HINTS 6 is a nationally representative survey routinely administered by the National Cancer Institute (NCI) [36]. The survey incorporated stratification and oversampling of rural and high minority urban strata to ensure it is nationally representative [36]. The National Cancer Institute employed a cross-sectional study design for this survey, collecting nationally representative data from non-institutionalized United States residents aged 18 years or older. The HINTS data focuses on health issues, including health information, social, telehealth, and genetic testing [36,37].

### 2.2. Data Collection

The data collection mode was a combination of paper-based and online questionnaires, available in both Spanish and English. The survey attained a household response rate of 28.1%, with 6252 participants, resulting in a weighted sample size of roughly 258,418,467 for population-level estimates [36]. This was conducted from 7 March 2022 to 8 November 2022. All respondents received a USD 2 pre-paid monetary incentive to encourage participation, and the current analysis of the HINTS 6 survey was limited to participants who responded to the question about perceived discrimination [36]. The final sample size was 6102. Other data collection, sample selection, weighting, and management information can be found on the following website: https://hints.cancer.gov/docs/methodologyreports/HINTS_6_MethodologyReport.pdf (accessed on 15 November 2024).

### 2.3. Dependent Variable

The dependent variable “perceived racial discrimination when getting medical care” was derived from the survey stem question C9, “Have you ever been treated unfairly or been discriminated against when getting medical care because of your race or ethnicity?”. A binomial response to this survey question was Yes (coded 1) and No (coded 0).

### 2.4. Independent Variables

The independent variables that were operationalized are education (less than high school, high school graduate, some college, and college graduate), household income (less than USD 20,000, USD 20,000 to <USD 35,000, USD 35,000 to <USD 50,000, USD 50,000 to <USD 75,000, and USD 75,000 or more), and rurality (rural/urban). Sociodemographic variables of interest were age groups (18–34 years, 35–49, 50–64, 65–74, and 75 years and older), race/ethnicity (non-Hispanic White, non-Hispanic Black/African American, Hispanic, non-Hispanic Asian, and non-Hispanic Other), and gender (male/female). The authors used the dataset owners’ categorization of these independent variables.

### 2.5. Data Analysis

The frequencies (unweighted) and weighted percentages were computed to describe the characteristics of the survey participants. Using a binomial logistic regression analysis, sampling weights were applied to assess the associations between the dependent variable and the listed independent variables. No multicollinearity was detected in the regression analysis. The goodness-of-fit test (Hosmer–Lemeshow test) indicated that the model provided a good fit. The STATA software v. 18 was used in the analysis, and *p* < 0.05 was evaluated as a statistically significant value.

## 3. Results

### 3.1. Descriptive Characteristics of the Participants

The mean age of respondents was 56 years. Ninety-two percent (92%) of the participants reported not being discriminated against when receiving medical care because of their race (Table 1). In total, 27% of the study participants were aged 50–64 years, 13% were aged 65–74, and 25% were aged 35–49. Moreover, 51% of the participants were women, and 61% identified their race as non-Hispanic White. A large percentage (88%) of the participants resided in urban areas, 33% held a college degree, and 44% earned USD 75,000 or more annually.

### 3.2. Sociodemographic Characteristics Associated with Perceived Discrimination When Receiving Medical Care in the United States

The results of the logistic regression analysis are presented in Table 2. Older adults aged 75 years and above had significantly lower odds of reporting perceived discrimination when receiving medical care compared to those aged 18–34 years (AOR = 0.24; 95% CI: 0.10–0.58). In terms of race and ethnicity, the odds of reporting perceived discrimination were significantly higher among non-Hispanic Blacks (AOR = 7.30; 95% CI: 4.48–11.88), Hispanics (AOR = 3.56; 95% CI: 2.45–5.17), non-Hispanic Asians (AOR = 5.95; 95% CI: 2.25–15.73), and individuals identifying as non-Hispanic Other (AOR = 10.91; 95% CI: 5.42–21.98), compared to non-Hispanic Whites. Conversely, compared to individuals from households earning less than USD 20,000, the odds of reporting perceived discrimination when receiving medical care were significantly lower among individuals from households earning between USD 50,000 and <USD 75,000 (AOR = 0.42; 95% CI: 0.23–0.78) and those earning USD 75,000 or more (AOR = 0.43; 95% CI: 0.22–0.83).

## 4. Discussion

The United States is highly diverse, with substantial disparities in health outcomes across different racial groups, socioeconomic statuses (SESs), ethnicities, and geographic locations, particularly in access to healthcare resources, necessitating ongoing attention for improvement [38]. The current study analyzed nationally representative data from the 2022 HINTS wave 6 to assess the individuals’ racial identity and other social, economic, and demographic dimensions linked with their perception of discrimination during episodes of medical care. Although 8% of study participants reported perceived racial discrimination when receiving medical care, the findings should be viewed in the context that the study participants who were non-Hispanic Black or African American also constituted a small proportion (11%) of the sample. Also, the data was collected after the pandemic, when some racial groups might have felt discriminated against.

Our primary independent variable, racial identity, emerged as one of the most significant predictors of perceived racial discrimination when receiving medical care. Our results show higher odds of perceived racial discrimination when receiving healthcare among Hispanics, non-Hispanic Asians, and non-Hispanic Other race identities; the odds ranged from 4 times higher to nearly 11 times higher in these minority groups compared to non-Hispanic Whites. Our study aligns with several studies, including research by Gee and Ford, demonstrating that non-Hispanic Black individuals are seven times more likely to report perceived discrimination in healthcare settings compared to non-Hispanic Whites [5]. The pandemic exacerbated the feeling of marginalization experienced by racial minorities, and the study findings that compared to non-Hispanic Whites, every other racial group in the analysis reported some level of racial discrimination when receiving medical care indicate a persistence in marginalization. Such discrimination in healthcare has profound implications for access to and utilization of healthcare, particularly in the context of preventive care services such as vaccination. For example, CarlLee et al. (2023) found that every 1-point increase in a racial discrimination score (0–45) was associated with a 1.03-fold increase in COVID-19 vaccine hesitancy [39]. The COVID-19 pandemic is amplifying health inequities for racial and ethnic minorities [40]. For instance, Lin et al. (2024) documented significant disparities in healthcare utilization, with non-Hispanic Whites more likely to access primary care physicians, specialists, and other healthcare providers compared to Hispanic, Asian, and Black populations [38]. These disparities persist even after accounting for socioeconomic status, highlighting the entrenched nature of racial inequities in healthcare access. Similarly, O’Neil et al. (2010) found that older African Americans in Maryland had higher hospitalization rates for ambulatory care-sensitive conditions compared to their White counterparts, even after controlling for demographic and socioeconomic factors [41]. Racial discrimination has a pervasive influence not only on how healthcare is delivered but also on how it is perceived and trusted by racially and ethnically marginalized communities.

Although ageism is rampant in the United States [42], our study showed a protective effect of age for older adults aged 75 years and older concerning their perceived discrimination when receiving medical care compared to those aged 18–34. The recent COVID-19 pandemic has brought attention to older people’s vulnerability. The COVID-19 pandemic has taken many older people’s lives and revealed ageism in a variety of contexts, for instance, discrimination in healthcare access, insufficient protection for the mental health of residents in assisted living facilities, and stereotyped media representations that set generations against one another. Ageism is a significant social health factor that has, until now, received little attention [43,44]. The authors considered the hypothesis that older adults have gotten used to racial discrimination and already accepted it as a normal way of life. Another hypothesis considered by the authors was that the vulnerability that comes with old age may make healthcare workers less discriminatory towards them.

Households with moderate incomes of USD 50,000 to <USD 75,000 and those with higher incomes of USD 75,000 or more also had a significantly lower risk of being discriminated against when receiving medical care than adults with the lowest household income of less than USD 20,000. Households with an income of at least USD 50,000 may be treated differently by healthcare workers because their income level makes them ineligible for government-assisted health insurance for low-income families. Research shows that individuals from lower-income backgrounds are more likely to perceive bias in the care they receive due to economic vulnerability and systemic disparities in healthcare interactions [45,46].

Gender and rurality were not significant predictors in the regression model. The authors postulated that both males and females likely experienced discrimination, which occurred regardless of their location. Gendered experiences of racism, especially while receiving medical care, will differ, and there is a need to explore this intersectionality.

These study findings provide scientific, actionable evidence that can strengthen policy and programmatic interventions aimed at reducing the occurrence of racial discrimination in medical care settings.

These findings can be best understood within the perspective of Ecosocial Theory, positing that health inequities and resulting disparities are not merely the product of random occurrences. They result as a byproduct of the intersectionality of social, political, and economic inequalities over the course of time [47]. By explaining how systemic racism, socioeconomic stratification, and differential access to resources become physiologically and behaviorally established, this paradigm helps to elucidate how the predictors in our models shape the health outcomes of individuals over their life course. The significantly greater odds of reported racial discrimination among minority groups that were identified in this study exemplify how structural racism occurs in the process of receiving healthcare services. The perceptions about race-related discrimination in healthcare are reflections of larger historical and institutional inequities. The Structural Racism Framework provides additional support for this interpretation. This framework highlights how macro-level systems, policies, and institutional practices in the healthcare industry systematically create disadvantages for racial and ethnic minorities [48]. Upstream system-level disadvantages or inequalities, in turn, lead to persistent downstream disparities in healthcare access, quality, and trust [49]. These frameworks, when considered as a whole, offer a crucial perspective that enables one to interpret alleged prejudice not as an individual anomaly but rather as a structural pattern deeply ingrained in the fabric of healthcare institutions.

This study’s findings should be interpreted keeping in view its limitations. First, this study used secondary data collected for purposes other than the specific research objective of the current study. This limitation may have resulted in the exclusion of several potential contextual predictors, such as immigration history and language barriers, confounders, and predictors of perceived discrimination during healthcare episodes, such as differences in staff characteristics, facility infrastructure, and care quality of healthcare providers. Secondly, the accuracy of the outcome variable may have varied by the time difference among respondents in the recency of care episodes relative to the time of HINTS 6. Furthermore, this survey collects self-reported data that has not been independently validated, which increases the risk of potential bias due to variations in individual patients’ interpretations of what constitutes discrimination, social desirability, and their ability to recall the level of discrimination they experienced. The causal relationships could not be ascertained because the research was based on a cross-sectional survey. Additionally, since this is the first time the question was asked in this survey, there is no longitudinal data for trend analysis. Despite these issues arising from secondary cross-sectional data, our study’s findings contribute a critical piece to the literature on the nature of related discrimination perceived by U.S. adults when receiving healthcare services. Future research directions: A crucial area for investigation is the role of community-based participatory studies in capturing the depth and nuances of individuals’ experiences with discrimination and inequities in healthcare. Such studies can provide rich, contextual insights into how policies can be designed and implemented to promote equitable access to healthcare resources and improve health outcomes for marginalized populations. Specific areas of focus should include the following: (1) A qualitative study that examines how discrimination based on country of origin, language barriers, and immigration status impacts healthcare access and quality. This includes understanding the unique challenges faced by immigrant populations and non-native English speakers in navigating the U.S. healthcare system. (2) Evaluating the role of healthcare organization mandatory anti-racist training on patient experiences and health outcomes. This involves creating evidence-based recommendations for policymakers to address racial discrimination in medical care and promote equitable access to healthcare.

The implications of these findings are profound. Perceived discrimination in healthcare settings can erode trust in medical institutions, reduce healthcare utilization, and exacerbate health disparities, particularly in the context of preventive care and chronic disease management. Addressing these inequities requires a multifaceted approach that includes policy reforms, targeted interventions, and a commitment to dismantling the structural barriers that perpetuate racial and ethnic disparities in healthcare.

## 5. Conclusions

This study contributes to the growing body of evidence highlighting the persistent and pervasive nature of racial and ethnic disparities in perceived discrimination within the U.S. healthcare system. Healthcare organizations should take proactive measures by mandating every health worker to take anti-racism training at least annually. Patients should be encouraged to provide feedback on their visit with providers anonymously, and providers with consistent patient complaints of racial discrimination should be sanctioned. Hiring diverse and qualified providers may make patients from other racial and ethnic backgrounds more comfortable during their healthcare visits. Additionally, it is crucial to involve diverse groups in the development of evidence-based public health interventions that are culturally responsive and tailored to the unique needs of marginalized populations.

As the United States continues to grapple with the legacies of systemic racism, public health researchers, policymakers, and healthcare providers must work collaboratively to create a more equitable healthcare system. This includes not only addressing the immediate manifestations of discrimination but also tackling the underlying structural factors that sustain these inequities by enacting legislations that promote health equity. Health equity can only be achieved when every individual has access to quality healthcare without fear of discrimination. Only through sustained and concerted efforts, grounded in collaboration and inclusivity, can we hope to dismantle structural barriers and achieve health equity for all.

## Figures and Tables

**Table 1:** Descriptive statistics of the study participants’ characteristics, 2022.

Variable	Frequency (N = 6102)	Weighted%
Dependent Variable		
Perceived Racial Discrimination		
No	5607	92%
Yes	495	8%
Independent Variables		
Age Groups (in years)		
18–34	939	26%
35–49	1240	25%
50–64	1772	27%
65–74	1356	13%
75+	847	9%
Gender		
Male	2307	49%
Female	3535	51%
Race/Ethnicity		
Non-Hispanic White	3203	61%
Non-Hispanic Black/African American	889	11%
Hispanic	1001	17%
Non-Hispanic Asian	288	6%
Non-Hispanic Multiple Races	184	5%
Urban/Rural Designation		
Urban	5441	88%
Rural	811	12%
Education Level		
Less than High School	387	7%
High School Graduate	1068	22%
Some College	1672	39%
College Graduate or More	2721	33%
Household Income		
Less than USD 20,000	959	14%
USD 20,000 to <USD 35,000	729	11%
USD 35,000 to <USD 50,000	732	12%
USD 50,000 to <USD 75,000	937	18%
USD 75,000 or More	2163	44%

**Table 2:** Logistic regression analysis of perceived racial discrimination when receiving medical care, 2022.

Sociodemographic Characteristics		AOR	95% Cl		Sig
			LL	UL	
Age (Ref. Category: 18–34 years)					
35–49 years		1.31	0.73	2.35	0.356
50–64 years		0.87	0.56	1.37	0.546
65–74 years		0.73	0.405	1.34	0.308
75 years and older		**0.24**	0.100	0.58	0.002
Gender (Ref. Category: Male)					
Female		1.08	0.68	1.71	0.739
Race/Ethnicity (Ref. Category: Non-Hispanic White)					
Non-Hispanic Black/African American		**7.30**	4.48	11.88	<0.001
Hispanic		**3.56**	2.45	5.17	<0.001
Non-Hispanic Asian		**5.95**	2.25	15.73	0.001
Non-Hispanic Other		**10.91**	5.42	21.98	<0.001
Educational Level (Ref. Category: Less than High School)					
High School Graduate		0.76	0.37	1.57	0.460
Some College		1.16	0.54	2.46	0.701
College Graduate/More		1.10	0.53	2.29	0.803
Rurality (Ref. Category: Rural)					
Urban		1.21	0.66	2.19	0.531
Household Income (Ref. Category: Less than USD 20,000)					
USD 20,000 to <USD 35,000		0.67	0.35	1.32	0.249
USD 35,000 to <USD 50,000		0.77	0.41	1.46	0.416
USD 50,000 to <USD 75,000		**0.42**	0.23	0.78	0.007
USD 75,000 or More		**0.43**	0.22	0.83	0.013

Note: Bolded adjusted odds ratios indicate statistically significant differences at *p* ≤ 0.05. Abbreviations: AOR—Adjusted Odds Ratio; CI—Confidence Interval; LL—Lower Limit; Ref. Category—Reference Category; Sig—Significant Level at *p* ≤ 0.05; UL—Upper Limit. The reference outcome category: Perceived racial discrimination–No.

## Data Availability

Publicly available datasets were analyzed in this study. This data can be found here (National Cancer Institute, Health Information National Trends Survey): https://hints.cancer.gov/data/download-data.aspx (accessed on 15 November 2024).
